# The Similar Effects of miR-512-3p and miR-519a-2-5p on the Promotion of Hepatocellular Carcinoma: Different Tunes Sung With Equal Skill

**DOI:** 10.3389/fonc.2020.01244

**Published:** 2020-08-07

**Authors:** Tao Rui, Xueyou Zhang, Shi Feng, Haitao Huang, Shaowei Zhan, Haiyang Xie, Lin Zhou, Qi Ling, Shusen Zheng

**Affiliations:** ^1^Division of Hepatobiliary and Pancreatic Surgery, Department of Surgery, School of Medicine, First Affiliated Hospital, Zhejiang University, Hangzhou, China; ^2^Collaborative Innovation Center for the Diagnosis and Treatment of Infectious Diseases, School of Medicine, The First Affiliated Hospital, Zhejiang University, Hangzhou, China

**Keywords:** C19MC, hepatocellular carcinoma, miRNA cluster, recurrence, therapy

## Abstract

Although the therapeutic methods of hepatocellular carcinoma (HCC) have made great advances, the current situation is that HCC is the common malignancy. Our previous bioinformatic study presented that two members of C19MC (mir-512-1 and mir-519a-2) acted as crucial roles in the HCC progression. In this study, we first demonstrated that the miR-512-3p and miR-519a-2-5p, which were spliced from the mir-512-1 and mir-519a-2, were the functional mature miRNAs. Meanwhile, both miR-512-3p and miR-519a-2-5p were significantly upregulated in human HCC samples and HCC cell lines. The miR-512-3p and miR-519a-2-5p promoted the proliferation, invasion, and metastasis *in vitro* and *in vivo*. Moreover, the two miRNAs co-targeted the downstream tumor suppressors MAP3K2 and MAP2K4 and subsequently achieved the HCC progression. In the clinical cohort, high expression of miR-512-3p and miR-519a-2-5p acted as two risk factors for HCC recurrence and distinguished patients with poor tumor-free survival after radical resection. The integration of the two miRNAs into the AJCC staging system significantly improved the accuracy for the prediction of HCC recurrence. Our study suggests that miR-512-3p and miR-519a-2-5p have similar effects on the promotion of HCC progression. They can be robust markers for the prediction of HCC recurrence and therapy targets.

## Introduction

Hepatocellular carcinoma (HCC) remains one of the major factors of tumor-related death worldwide (1). In the past two decades, the approaches to prevention (2), detections (3), early diagnoses (4), and multidisciplinary therapy (5) of HCC have been notably evolved. Besides, the advancement of pharmacological treatment with targeted agents has significantly prolonged the overall survival of advanced HCC (6–8). However, the incidence and HCC-related mortality continue to increase, and the advanced HCC accounts for a large proportion (9). Hence, to further explore the mechanism of HCC development and search for therapeutic targets is urgent.

An extensive literature has reported that miRNAs involved in tumorigenesis and tumor progression (10). These small fragments of RNAs regulate tumor progression by inhibiting transcription of target genes or by degrading target mRNA. However, single miRNA is often feeble and unsteady in the clinical trial of cancer therapy. Thus, a combination of miRNAs with similar effects from more than 800 human-derived and identified miRNAs is anticipated (11).

miRNA cluster is defined as a family of physically adjacent miRNAs that are transcribed from nearby genomic regions, and their transcriptional regulatory mechanisms are usually analogous (12). Moreover, the miRNAs generally show phylogenetically identical or similar seed sequences (13, 14). Indeed, several cases reveal that even miRNA clusters with different seed sequences can achieve sequential amplification effect by inhibiting the targets belonging to the same pathway (15, 16). Therefore, the origin and characteristics of miRNA clusters show that they constantly exhibit similar regulation activity

As the largest miRNA cluster that has been identified, chromosome 19 microRNA cluster (C19MC) contains a group of 59 mature miRNAs (17). Some sporadic reports present the role of individual members of C19MC in tumorigenesis (18, 19). Previously by digging The Cancer Genome Atlas (TCGA) database and bioinformatics analysis, the expression of C19MC was comprehensively upregulated in HCC. 37 (80.4%) and 45 (97.8%) miRNAs from C19MC belonged to the top 50 and top 100 differentially upregulated miRNAs. And the mir-512-1 and mir-519a-2 derived from C19MC were proposed, due to their potential similar effects: (i) the upregulation of these two miRNAs was positively correlated with the tumor burden, tumor stage, and tumor grade. (ii) the high expression of the two miRNAs was positively correlated with the poor survival of HCC (20).

In this study, we assessed that the mir-512-1 and mir-519a-2 spliced into two functional mature miRNAs, miR-512-3p and miR-519a-2-5p, which were upregulated in HCC. The similar effects of the miR-512-3p and miR-519a-2-5p on the promotion of the proliferation, invasion, and metastasis in HCC *in vitro* and *in vivo* were detected. The MAP3K2 and MAP2K4 were screened and confirmed to be the co-targets of the two miRNAs. At last, we proved that the miR-512-3p and miR-519a-2-5p promoted the early recurrence of HCC.

## Materials and Methods

### Patients and Specimens

HCC and the matched adjacent non-tumor tissues were obtained from the patients who underwent the hepatectomy in the First Affiliated Hospital, College of Medicine, Zhejiang University, China from April 2015. All patients were confirmed as HCC by postoperative histopathology. This research protocol was approved by the Ethical Committee of the First Affiliated Hospital, School of Medicine, Zhejiang University.

### Cell Culture

The HCC cell lines, SNU-449 which was purchased from American Type Culture Collection, was cultured in Roswell Park Memorial Institute 1,640 medium (GIBCO, USA) and Eagle's minimum essential medium (Thermo Fisher Scientific, USA), respectively. Besides, HUH-7, LM3, and L02, which were purchased from the Cell Bank Of Chinese Academy of Sciences, were cultured in Dulbecco's modified Eagle's medium (Thermo Fisher Scientific, USA). Both media were supplemented with 10% fetal bovine serum (BioIND, China). All the cell lines were cultured at 37°C with 5% CO_2_ in a humidified incubator.

### Cell Transfection

The miR-512-3p mimics (Cat.miR10002823-1-5), miR-519a-2-5p mimics (cat.miR1180420011719) and the negative mimics control (cat.miR01102), miR-512-3p inhibitor (miR20002823-1-5), miR-519a-2-5p inhibitor (cat.miR1180420011719), and the negative inhibitor control (cat.miR02101) were synthesized from RiboBio. The specific siRNAs for silencing the expression of MAP3K2 and MAP2K4 were also synthesized from Ribobio. The RNA oligonucleotides were efficiently transfected into SNU-449 and HUH-7 by using the riboFECT™ CP (Ribobio, China).

Between the site of XhoI and KpnI, the Coding Sequence of MAP3K2 and MAP2K4 were cloned into plasmid GV230 (Genechem, China) for constructing the MAP3K2 and MAP2K4 expression vectors, respectively. Then the purified MAP3K2 and MAP2K4 plasmids were transfected into SNU449 and HUH-7 by using the Lipofectamine 3,000 (Invitrogen, USA).

For miR-512-3p or miR-519a-2-5p stable expression, lentiviral particles (Genepharma, China) were produced by the third-generation packaging system vectors- pGag/Pol, pRev, pVSV-G. The 293T cells were transfected with the vectors on the 10-cm culture dish. The supernatants containing the lentiviral particles were collected, filtered, and concentrated every 24 h. Then, after LM3 cells were transduced with miR-512-3p or miR-519a-2-5p overexpression lentiviral particles an MOI of 50 for 24 h, 2 μg/mL of puromycin (Thermo Fisher Scientific, USA) was used to screen for stable expression of miRNAs.

### Quantitative Reverse-Transcription PCR (qRT-PCR) for mRNA and miRNA

Total RNA, including mRNA and miRNA, was extracted from tissues and cell lines by using TRIzol reagent (Invitrogen, USA). For mRNA reverse transcription, The total RNA was used to synthesize the cDNA according to the manufacturer's instructions of RevertAid First Strand cDNA Synthesis Kit (Thermo Fisher Scientific, USA). The real-time PCR amplification was performed by using FastStart Universal SYBR Green Master (Roche, Switzerland), in accordance with the manufacturer's protocols. GAPDH was used as the target gene normalization control. The primers sequences for mRNA amplification are listed in Table S1.

Stem-loop miRNA qRT-PCR was performed for highly specific detection of miRNA expression by using Bulge-Loop miRNA qRT-PCR Starter Kit (Ribobio, China). The specific reverse-transcription primers and PCR amplification primers (miR-512-3p, cat.MQPS0001712-1-100; miR-512-5p, cat.MQPS0001713-1-100; miR-519a-3p, cat.MQPS0001754-1-100; miR-519a-2-5p, cat.MQP-0101) for detecting the miRNA amplification were designed and purchased from Ribobio. According to manufacturer's instructions, the synthesis of the miRNAs cDNA was performed with the condition of 42°C for 60 min and 70°C for 10 min, and the PCR amplification was performed with the condition of 40 cycles at 95°C for 15 s and 60°C for 30 s. U6 (cat.ssD0904071008 Ribobio, China) was used as the miRNA normalization control. The qRT-PCR was performed in triplicate and the relative expression levels were calculated with the 2^−ΔΔ*Ct*^ method.

### Western Blot Analysis

Total proteins from HCC cell lines were extracted by using RIPA lysis buffer (BestBio, China). The concentration of the proteins was quantified by using the BCA kit (Beyotime, China) according to the manufacturer's instructions. Then the lysates were electrophoresed by using SDS-PAGE in 10% Bis-Tris gel (Thermo Fisher Scientific, USA) and transferred onto polyvinylidene difluoride membranes. After being blocked, the membranes were incubated with primary antibody against MAP3K2 (cat.19607, Cell Signaling Technology, USA) (80kDa); MAP2K4 (cat. 9152, Cell Signaling Technology, USA) (55kDa) overnight at 4°C. The secondary goat-anti-rabbit GAPDH antibody (cat.FD0063, Fudebio, China) (36kDa) was incubated for 1 h at room temperature. The immunoreactive proteins were detected with Enhanced chemiluminescence (Fudebio, China).

### Immunohistochemical Staining

Three tumor samples from the sacrificed mice were randomly chosen and embedded in paraffin. The Immunohistochemical staining was performed as described before (21). The tissue sections were incubated with primary antibody against MAP3K2 (cat.db7008, Diagbio, China), MAP2K4 (cat.A7724, ABclonal, China), Ki67 (cat.db901, Diagbio, China) at 4°C overnight and then incubated with secondary HRP anti-rabbit antibody (Servicebio, China). Then the sections were stained with diaminobenzidine and further counterstained with hematoxylin. Five images were randomly captured from the immunoreactive protein of each section and quantified with the Image J Software (Version 1.52) (National Institutes of Health).

### Dual-Luciferase Reporter Assay

The wild-type (WT) or mutant type (Mu) 3′ untranslated region (3′UTR) sites of MAP3K2 or MAP2K4 were synthesized and constructed into pGL3 vector (Promega, USA), respectively. After 293T cells (1 × 10^4^) were seeded in 96-well plates for 24 h, they were co-transfected with MAP3K2 or MAP2K4 luciferase vector, and miRNA mimics or miRNA controls, respectively. After 48 h, the Dual-Luciferase Reporter Assay System (Promega, USA) was performed to detect the relative luciferase activity according to the manufacturer's instructions.

### Cell Proliferation Assay

Cell Counting Kit-8 (CCK-8) assay and 5-Ethynyl-2′-deoxyuridine (EdU) assay were used for detecting cell proliferation. After HCC cell lines were seeded in the 96-well plates (For CCK8) or 24-well plates (for EdU) for 24 h, the synthesized miRNA oligonucleotides were transfected as above, respectively. The CCK-8 reagent (Dojindo Molecular Technologies, Japan) was added and incubated for 1 h, the absorbance at 450 nm was detected by the spectrophotometric reader (Multiskan FC, Thermo Scientific). The Click-iT EdU Alexa Fluor 488 Assay Kit (Invitrogen, USA) was used to measure cell proliferation according to the manufacturer's instructions.

### Cell Invasion Assay

The transwell system (Corning Incorporated, USA) was used to cell invasion assay. Briefly, HCC cells were transfected with miRNA oligonucleotides for 24 h, respectively. Then, the Matrigel (BD Biosciences, USA) was coated into the upper chamber of the corning transwell membrane. After the chambers were incubated with serum-free medium for 2 h, the transfected cells were resuspended into the upper chamber and the lower chamber of the plate was contained with complete culture media. After cultivation for 24 and 48h, the chambers were fixed with paraformaldehyde and stained with crystal violet (Sigma-Aldrich, USA). The stained cells were counted to assess the invasiveness.

### Tumor Model

The Balb/c male nude mice (6–8 weeks) were obtained from Shanghai Experimental Animal Center, Chinese Academy of Science, and received care according to the criteria of the National Institute Guide for the Care and Use of Laboratory Animals (Chinese version of 8th guide). Before the mice were injected with cells, they were randomly assigned to each group. To minimize the suffering of mice, all of them were anesthetized and euthanized with CO_2_ inhalation before sacrifice. The animal research protocol was approved by Ethical Committee of the First Affiliated Hospital, School of Medicine, Zhejiang University ([2017]073).

The LM3 cell lines (2 × 10^6^) that have been screened for stable expression of miR-512-3p, miR-519a-2-5p, and their controls were subcutaneously injected into the right flank of the mice (6 weeks, *n* = 6 in each group). After the mice xenograft tumor burden models were established, the tumor volume (V) was assessed twice a week by measuring the length (L) and width (W) of the tumor (V= W^2^ × L/2). After 30 days, the mice were sacrificed, and all tumor specimens were collected for cryopreservation or fixed preservation.

For further evaluating the invasion and metastasis capacity *in vivo*, the orthotopic HCC mouse models (8 weeks, *n* = 5 in each group) were used. Briefly, after being anesthetized (pentobarbital) and sterilized, the upper abdomen of mice were open through the upper abdominal incision, and carefully expose the liver. The miR-512-3p, miR-519a-2-5p, or their controls stable expression LM3 cell lines (1 × 10^6^, 1:1 in matrigel, 50 μl) were grafted in the right lobe of the mice liver, respectively. Then the surgical incision was conventional closed. After 5 weeks, the invasion and metastasis were evaluated with the living bioluminescence imaging.

### Statistical Analysis

Quantitative variables were described as the mean ± standard deviation (SD) or median ± interquartile range (IQR). A comparison of the quantitative variables between the two groups was performed with Student's *t*-test or Mann–whiney test, and one-way ANOVA followed by *post hoc* Bonferroni test was used among more than two groups. Categorical variables were performed with Pearson's χ^2^-test or Fisher's exact test. Kaplan–Meier survival curves were assessed with the log-rank test. The univariate Cox proportional hazards regression analysis was used to screen the risk factors of HCC recurrence. The area under receiver operating characteristics (AUROC) was performed to predict the recurrence of HCC. Statistical analysis was performed with SPSS Software (Version 19.0) and *p* < 0.05 was set as the significance level. “*,” “**,” “***,” and “****” represented the *p* < 0.05, 0.01, 0.001 and 0.0001, respectively.

## Results

### The Upregulated miR-519a-2-5p and miR-512-3p, Which Were Originated From the Two Oncogenic mir-519a-2 and mir-512-1, Could Be Ideal Research Candidates on HCC

**Figure 1:**
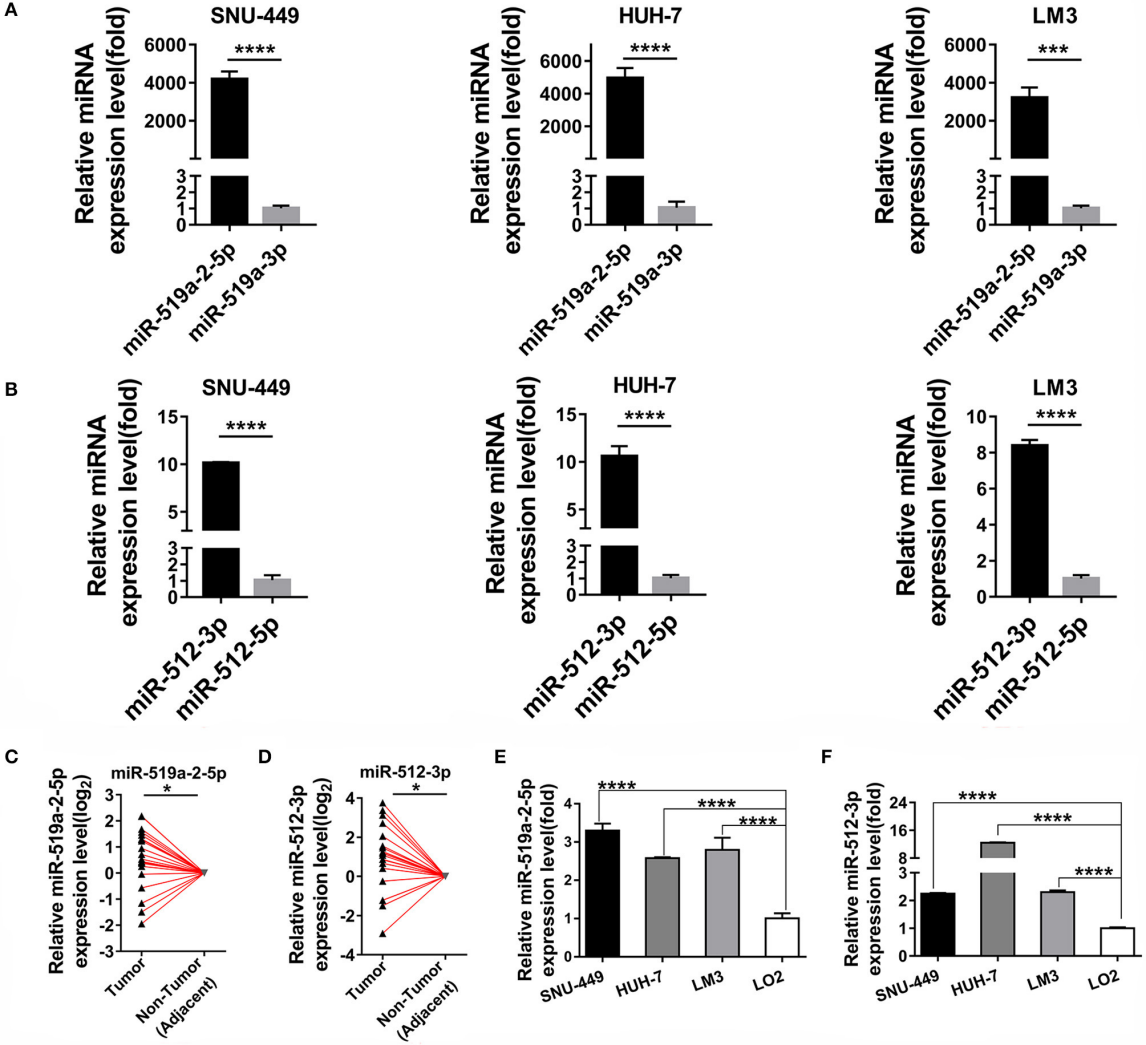
Two miRNA candidates expression levels between hepatocellular carcinoma (HCC) and adjacent non-tumor tissues. **(A,B)** The expression levels of miR-519a-2-5p **(A)** and miR-512-3p **(B)** were upregulated, compared with their complimentary chains (miR-519a-3p and miR-512-3p) from three HCC cell lines. The statistics analysis was performed with the Mann–Whitney test. **(C,D)** The expression levels of miR-519a-2-5p **(C)** and miR-512-3p **(D)** were significantly upregulated in 20 paired HCC tissues, compared with that in paired adjacent non-tumor tissues. The expression levels in adjacent non-tumor tissues were normalized and set as 0. The *P*-values were calculated with the Student's paired *t*-test. **(E,F)** The expression levels of miR-519a-2-5p **(E)** and miR-512-3p **(F)** in three HCC cell lines the LO2. The statistics analysis was performed with the one-way ANOVA test. “*”, “***” and “****” represented the *p*-value < 0.05, 0.001 and 0.0001, respectively.

As described above, by digging the TCGA database previously, the two members of C19MC, mir-519a-2 and mir-512-1, acted as the potential key agents that advanced the HCC tumor burden, tumor stage, and promoted poor prognosis (20). We further explored their respective functional mature miRNAs originated from mir-519a-2 and mir-512-1. The results suggested that the miR-519a-2-5p and miR-512-3p were highly expressed, compared with their complementary strands (miR-519-3p and miR-512-5p), respectively (Figures 1A,B). Therefore, it indicated that miR-519a-2-5p and miR-512-3p could be the functional mature miRNAs from the mir-519a-2 and mir-512-1. Then the two mature miRNAs were concentrated on in this experiment. Firstly, the expression levels of miR-519a-2-5p and miR-512-3p in HCC were evaluated in our Chinese patient cohorts. From 20 pairs of randomly selected HCC tissues, the miR-519a-2-5p and miR-512-3p were significantly up-regulated in HCC, compared to the matched adjacent non-tumor tissues (Figures 1C,D). Furthermore, the expression levels of the two miRNAs were verified between the three HCC cell lines (SNU-449, HUH-7, and LM3) and immortalized liver cell line (LO2). These two miRNAs showed significant expression elevation in three HCC cell lines compared to LO2 cells (Figures 1E,F). As a result, we confirmed miR-519a-2-5p and miR-512-3p as robust research candidates and further validated their effects on HCC.

### miR-519a-2-5p and miR-512-3p Promoted HCC Proliferation and Invasion *in vitro*

To detect the effects of miR-519a-2-5p and miR-512-3p on the proliferation of HCC *in vitro*, miRNA mimics and inhibitor oligonucleotides, as well as their scrambled controls, were used for HCC cell lines transfection. After SNU-449, HUH-7 were transfected with the corresponding miRNAs mimics, qPCR confirmed that the expression levels of miR-519a-2-5p and miR-512-3p were sharply overexpressed among the three HCC cell lines (Figure S1A).

**Figure 2:**
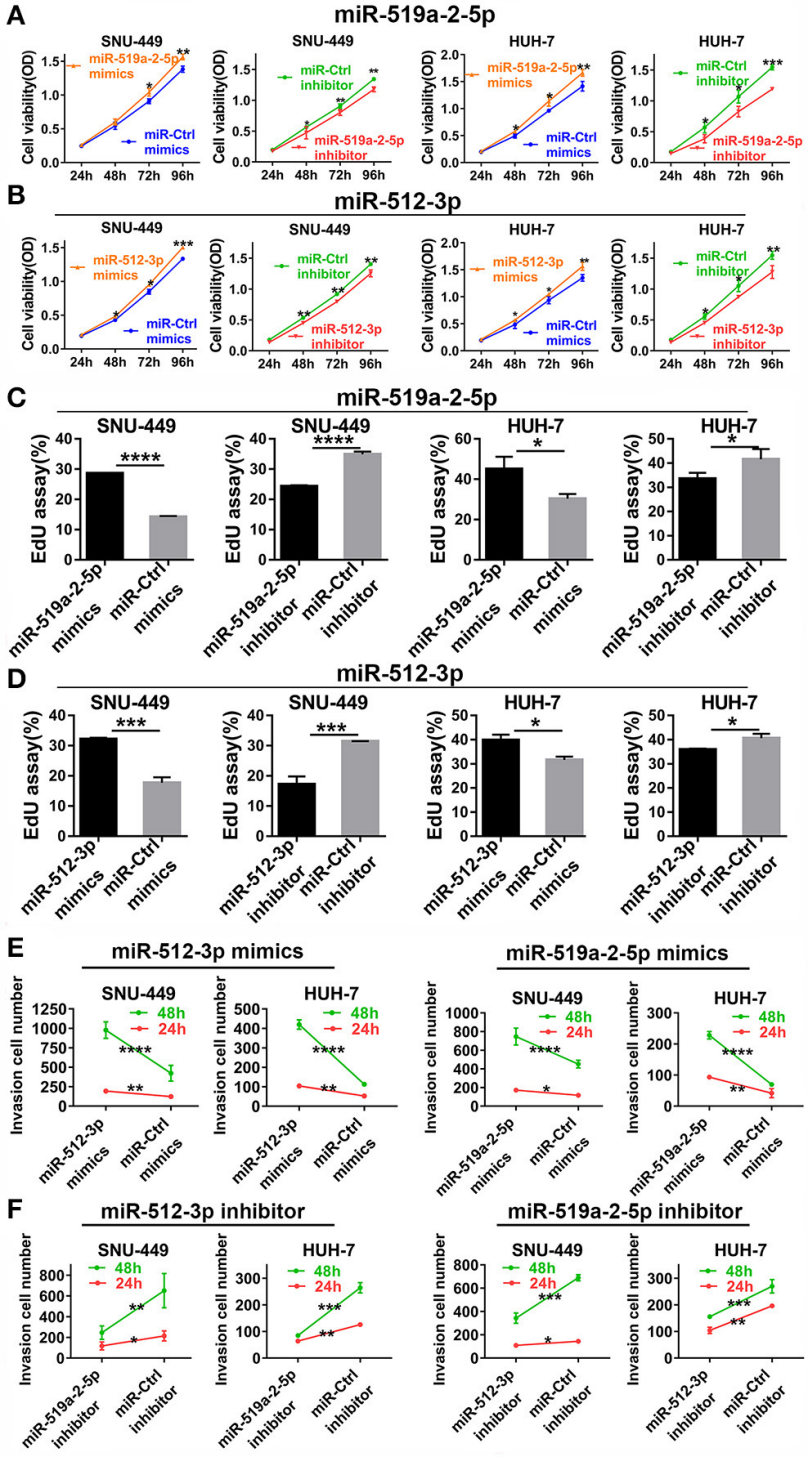
MiR-519a-2-5p and miR-512-3p promoted hepatocellular carcinoma proliferation and invasion *in vitro*. **(A,B)** CCK-8 assay detected the change of HCC cell proliferation after transfected with miR-519a-2-5p **(A)** or miR-512-3p **(B)** mimics, inhibitor, and their controls. **(C,D)** EdU assays showed the effects of miR-519a-2-5p **(C)** or miR-512-3p **(D)** on HCC cells after transfected with the mimics, inhibitor, or their controls, respectively. **(E,F)** Line charts showed that the invasion capacity (at time of 24 and 48 h) of HCC cells was changed after transfected with miR-519a-2-5p **(E)** or miR-512-3p **(F)** mimics, inhibitor, or the controls. The statistics analysis was performed with Student's *t*-test. “*”, “**”, “***” and “****” represented the *p*-value < 0.05, 0.01, 0.001 and 0.0001, respectively.

After successful oligonucleotides-transfection, the effects of these two miRNAs on the ability of HCC cell proliferation were detected. Up-regulation of miR-519a-2-5p and miR-512-3p could promote the proliferation of HCC cells by CCK8. The opposite results were acquired from the miR-519a-2-5p and miR-512-3p inhibitor-transfected cells (Figures 2A,B). Besides, the EdU assay also assessed that the viability of HCC cells could be increased by the two miRNA mimics and decreased by the two miRNA inhibitors (Figures 2C,D). Meanwhile, the transwell matrigel assay at the time points of 24 and 48 h were performed to assess the effects of miR-519a-2-5p and miR-512-3p on the HCC invasion. The results confirmed that overexpression of miR-519a-2-5p and miR-512-3p dramatically promoted the invasiveness of SNU-449 and HUH-7 through the Matrigel-coated chamber compared with control groups (Figure 2E, Figures S2A,B). Meanwhile, after transfected with the miR-519a-2-5p and miR-512-5p inhibitor, the invasiveness of SNU-449, HUH-7 cells was significantly reduced (Figure 2F, Figures S3A,B). These phenomena were confirmed at both the time points of 24 and 48 h and were especially noticeable at the time point of 48 h.

### miR-519a-2-5p and miR-512-3p Promoted HCC Metastasis and Growth *in vivo*

**Figure 3:**
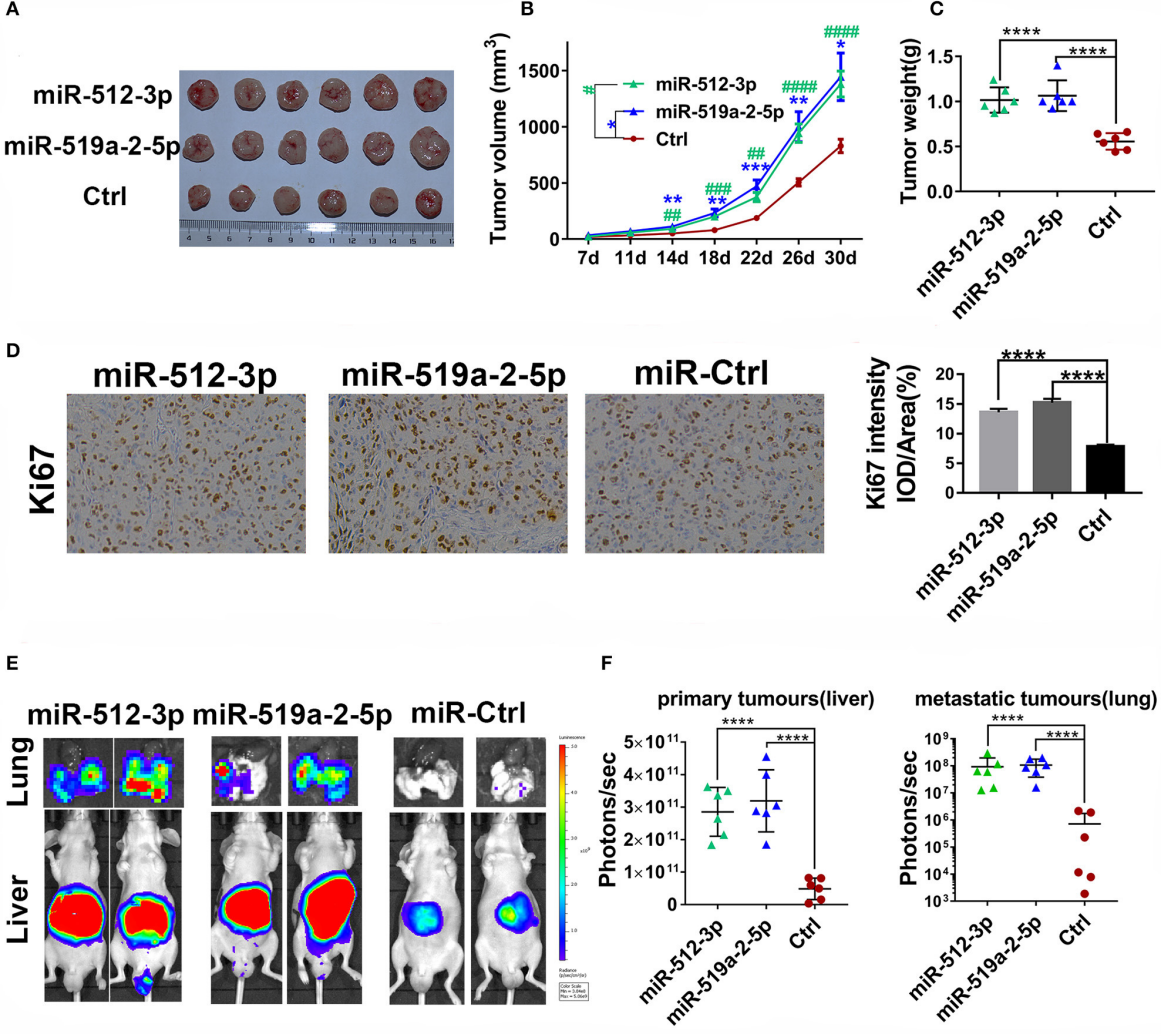
MiR-519a-2-5p and miR-512-3p promoted HCC metastasis and growth *in vivo*. **(A–C)** The efficiency of miR-512-3p and miR-519a-2-5p on the proliferation of HCC xenograft tumors. **(A)** The images of the xenograft tumors which were derived from miR-512-3p, miR-519a-2-5p, and the control-transfected LM3. **(B)** Tumor volume was measured. “*” represented the comparison between the miR-519a-2-5p group and the control group, “#” represented the comparison between the miR-512-3p group and the control group. **(C)** Tumor weight was measured on the day of 30. **(D)** The immunohistochemical staining of ki-67 from the tumors tissues derived from miR-519a-2-5p, miR-512-3p-transfected LM3, and their controls. **(E,F)** The efficiency of miR-512-3p and miR-519a-2-5p on the invasion and metastasis of HCC orthotopic mouse models. **(E)** Representative bioluminescence imaging of abdominal tumor burden and lung metastasis in all groups. **(F)** Quantification of bioluminescent signal intensity on the liver (primary tumors) and lungs (metastatic tumors). All error bars indicated the mean ± SD. The statistics analysis was performed with the one-way ANOVA test. “**”, “***” and “****” represented the *p*-value < 0.01, 0.001 and 0.0001, respectively. “##” “###” “###” represented the *p*-value < 0.01, 0.001 and 0.0001, respectively.

To detect the effects of miR-519a-2-5p and miR-512-3p on HCC *in vivo*, the xenograft models in nude mice were generated. After the LM3 cells were transfected with constructed overexpression lentiviral particles and further selected with puromycin, the miR-519a-2-5p or miR-512-3p stably expressed LM3 cells were obtained (Figure S1B). Each mouse was implanted with the corresponding cells and sacrificed after 30 days (*n* = 6 in each group). The results showed that both miR-519a-2-5p and miR-512-3p transfected LM3 achieved rapid growth *in vivo* (Figure 3A). Compared with the control group, the tumor volume (Figure 3B) and tumor weight (Figure 3C) of both the miR-519a-2-5p and miR-512-3p transfected mice were significantly larger. The cell-proliferation markers, Ki-67, was detected using immunohistochemical staining on the xenograft tumors. The results showed a greater accumulation of Ki-67 in miR-519a-2-5p and miR-512-3p transfected groups than that in controls (Figure 3D).

To further assess the ability of the two miRNAs on promoting HCC metastasis, the *in situ* HCC models were used (*n* = 5 in each group). The living bioluminescence imaging signal of the liver and lung was detected for assessing the tumor invasion and metastasis. Given that these two miRNAs significantly promoted the invasion of HCC, this experiment focused on the phenomenon of HCC metastasis to distant areas such as lung metastasis. The results showed that these two miRNAs not only promoted the invasion of HCC on the liver but also promoted metastasis on the lung (Figure 3E). The bioluminescence imaging signal of liver and lung in both miR-519a-2-5p and miR-512-3p transfected groups were much higher, compared with the control group (Figure 3F). Higher frequency and hyperintense signal of lung metastasis occurred in the miR-519a-2-5p (100%, 6/6) and miR-512-3p (100%, 6/6) transfected groups, while only two mice (33%, 2/6) in the control group were captured with weak signals in the lung. Therefore, miR-519a-2-5p and miR-512-3p promoted HCC lung metastasis with a high tumor burden, which was consistent with the high invasion state of the tumor on the liver.

### miR-519a-2-5p and miR-512-3p Co-targeted the Expression of MAP3K2 and MAP2K4

Given that miR-519a-2-5p and miR-512-3p exhibited similar effects on HCC, a hypothesis was raised that the two miRNAs might inhibit the common targets. To confirm the hypothesis, the common potential targets of the two miRNAs were screened and overlapped, by the online target database-miRWalk (http://mirwalk.umm.uni-heidelberg.de/) (Figure S4A). The common targets were performed with Kyoto Encyclopedia of Genes and Genomes (KEGG) enrichment. The results showed that the MAPK signaling pathway enriched the most targets, and within higher statistical significance and rich factors (Figure S4B). The targets in the MAPK pathway were screened with the criteria of low expression levels and poor prognosis prediction, by the online database-GEPIA (http://gepia.cancer-pku.cn/index.html). The results presented that only the MAP3K2 and MAP2K4 matched the criteria. The MAP3K2 and MAP2K4 were downregulated in HCC (Figure S4C). And the decrease of the MAP3K2 and MAP2K4 were positively correlated with the poor survival of HCC (Figure S4D). The MAP3K2 and MAP2K4 were also at the core of the targets construction network, after the targets in MAPK pathways were performed with PPI (protein-protein interaction) (Figure S4E). After reviewing the literature, we found that both two targets were the JNK axis of the MAPK pathway (22). All the results indicated that the MAP3K2 and MAP2K4 were the potential co-targets of the two miRNAs.

**Figure 4:**
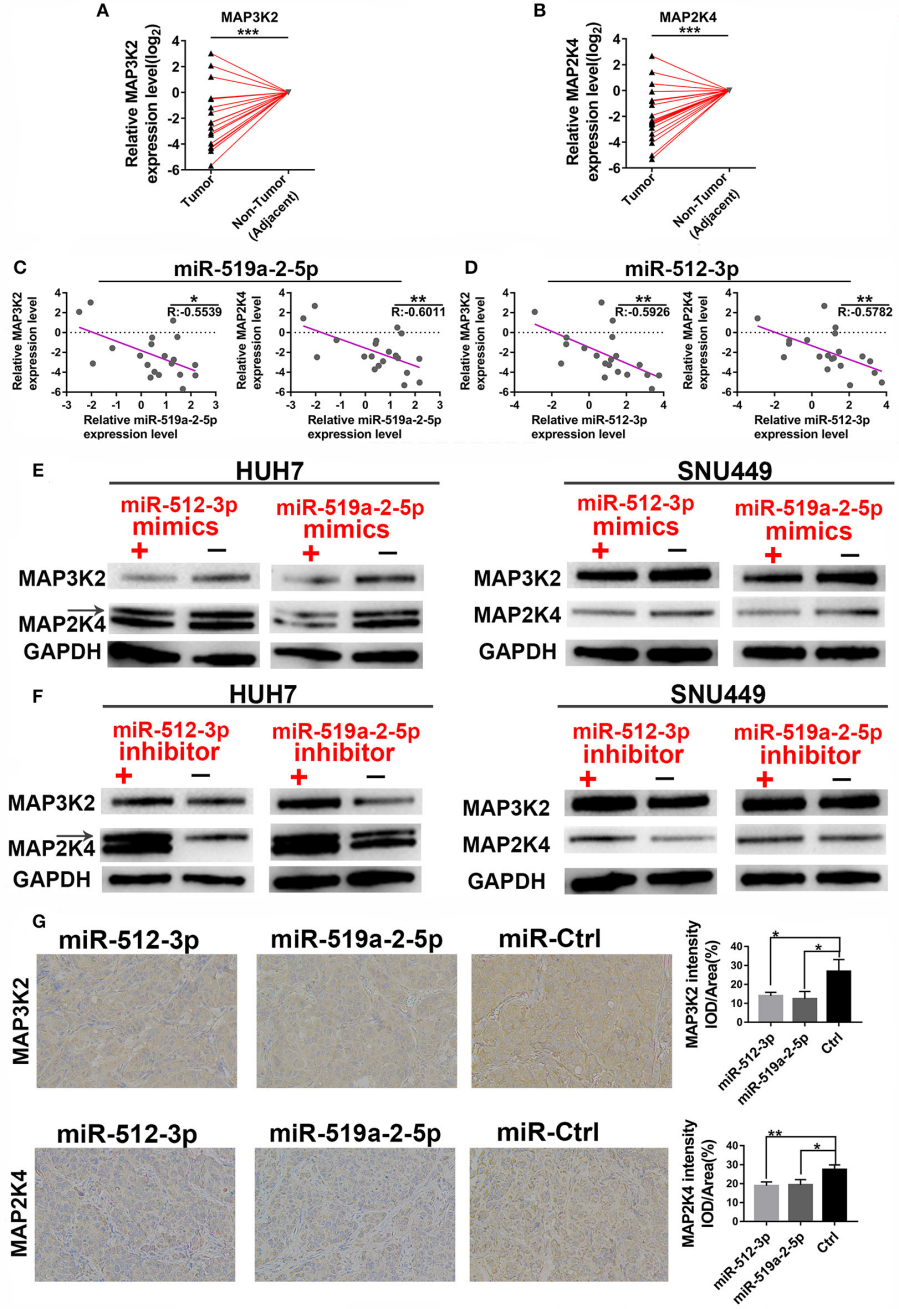
MiR-519a-2-5p and miR-512-3p co-targeted the expression of MAP3K2 and MAP2K4. **(A,B)** The expression of MAP3K2 (left) and MAP2K4 (right) in 20 paired HCC and adjacent non-tumor tissues. The expression levels in adjacent non-tumor tissues were normalized and set as 0. *P*-values were calculated with the Student's paired *t*-test. **(C,D)** The inverse correlation between the expression level of miR-519a-2-5p **(C)** and miR-512-3p **(D)** with MAP3K2 and MAP2K4. “*R*” represented the Pearson correlation coefficient. **(E,F)** Western blot analysis of the MAP3K2 and MAP2K4 expression in the HUH-7 and SNU-449 which were transfected with miR-519a-2-5p or miR-512-3p mimics (upper layer), inhibitor (lower layer) and their controls, respectively. **(G)** The immunohistochemical staining of MAP3K2 (upper layer) and MAP2K4 (lower layer) from the tumors tissues derived from miR-519a-2-5p, miR-512-3p-transfected LM3, and their controls. The statistics analysis was performed with the one-way ANOVA test. “*”, “**” and “***” represented the *p*-value < 0.05, 0.01 and 0.001, respectively.

We further performed the experimental methods to prove the results of the bioinformatics analysis above. Firstly, the expression levels of the two targets were detected to determine whether they were down-regulated. As expected, compared with the adjacent non-tumor tissues, both the MAP3K2 and MAP2K4 expression levels showed a prominent decrease in 20 HCC tissues (Figures 4A,B), with significantly negative correlations to miR-519a-2-5p and miR-512-3p (Figures 4C,D). Moreover, After SNU-449 and HUH-7 were transfected with mimics of the two miRNAs for 72 h, MAP3K2 and MAP2K4 protein were significantly down-regulated in the miRNA-mimics groups (Figure 4E), respectively. Meanwhile, the two miRNAs inhibitor indeed upregulated the expression levels of MAP3K2 and MAP2K4 (Figure 4F). Immunohistochemical staining also revealed that the lower MAP3K2 and MAP2K4 expression in the tumors formed by miR-519a-2-5p and miR-512-3p transfected LM3 cells, compared to the controls (Figure 4G).

**Figure 5:**
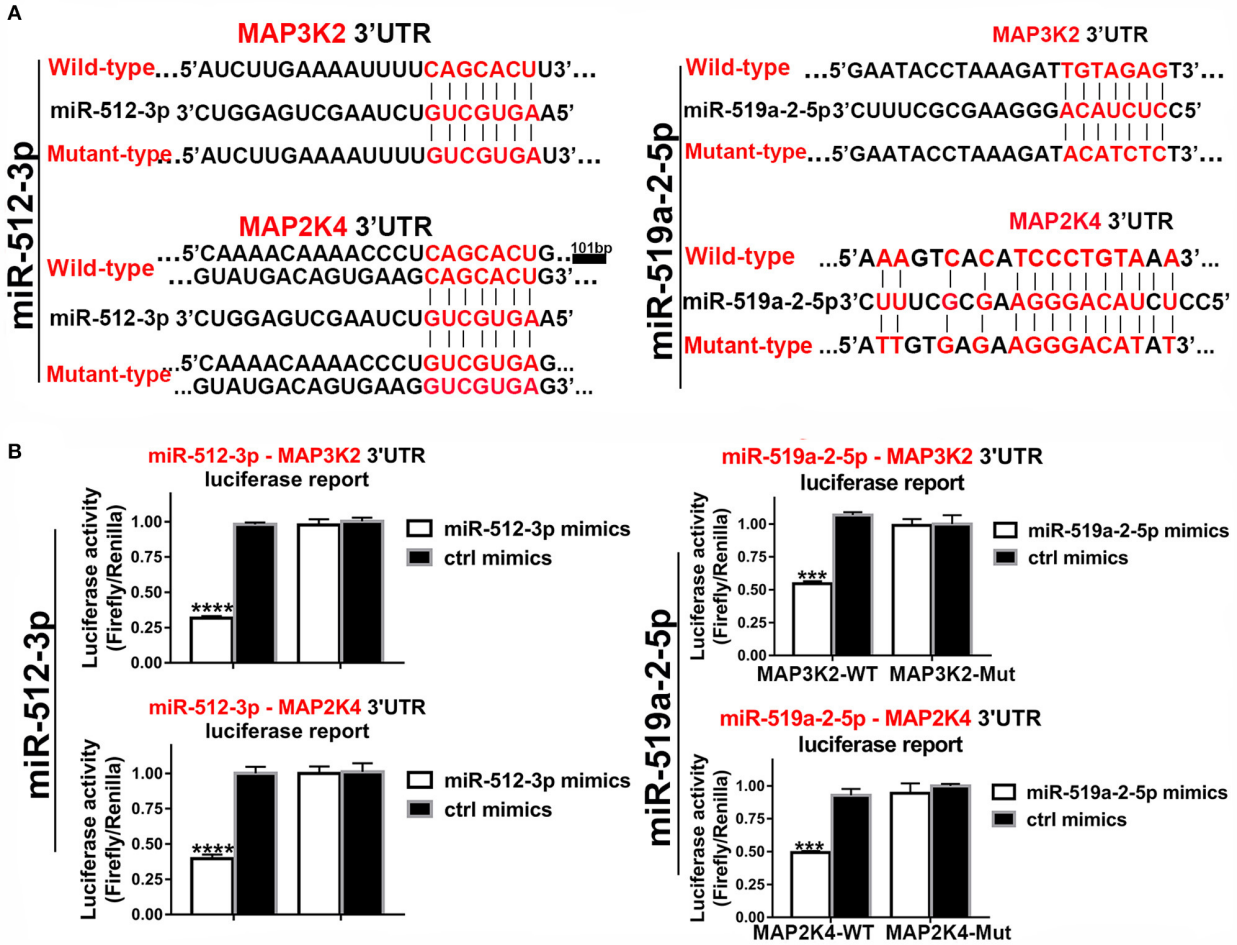
Luciferase assays confirmed the 3, UTR sites of MAP3K2 and MAP2K4 targeted by miR-512-3p and miR-519a-2-5p. **(A)** The predicted binding site sequence in the 3′UTR of MAP3K2 and MAP2K4 and the Corresponding mutated site. **(B)** Dual-luciferase assays showed the relative luciferase activities (Firefly/Renilla) among the HEK-293T cells co-transfected with miR-512-3p (left), miR-519a-2-5p (right) or control mimics and luciferase wild-type or mutant reporters. The statistics analysis was performed with the one-way ANOVA test. “***” and “****” represented the *p*-value < 0.001 and 0.0001, respectively.

In the 3′UTR of the MAP3K2 and MAP2K4 exist potential binding sites of the two miRNAs. Luciferase reporter assay was used to validate that MAP3K2 and MAP2K4 were the direct targets of the two miRNAs. The luciferase reporter vectors containing 3′ UTR binding sites and mutant counterpart were constructed (Figure 5A). By co-transfected with mimics, the luciferase activity was significantly inhibited with the wild-type 3′ UTR vector, while not with the mutant counterpart (Figure 5B). All the evidence proved that MAP3K2 and MAP2K4 were the direct targets of the two miRNAs.

The rescue experiments were performed to assess that the miRNA-induced functions were attributed to the two targets. The co-transfection of miRNA mimics and target gene overexpression plasmids harvested significant shreds of evidence. The results showed that both miR-512-3p and miR-519a-2-5p mimics improved the invasiveness of SNU-449 and HUH-7 cells, and those phenotypes were significantly rescued by both MAP3K2 and MAP2K4 overexpression after co-transfection (Figure S5). Then the effects of miRNA inhibitor and target genes siRNA co-transfection were also evaluated. One of the most effective siRNA from three siRNA candidates was significantly selected to knock down the expression of MAP3K2 or MAP2K4, respectively (Figure S6). Both of MAP3K2 and MAP2K4 knockdown dramatically reversed the decrease of invasiveness duo to the two miRNA inhibitor (Figure S7).

### The miR-519a-2-5p and miR-512-3p Promoted the Early Recurrence of HCC

**Figure 6:**
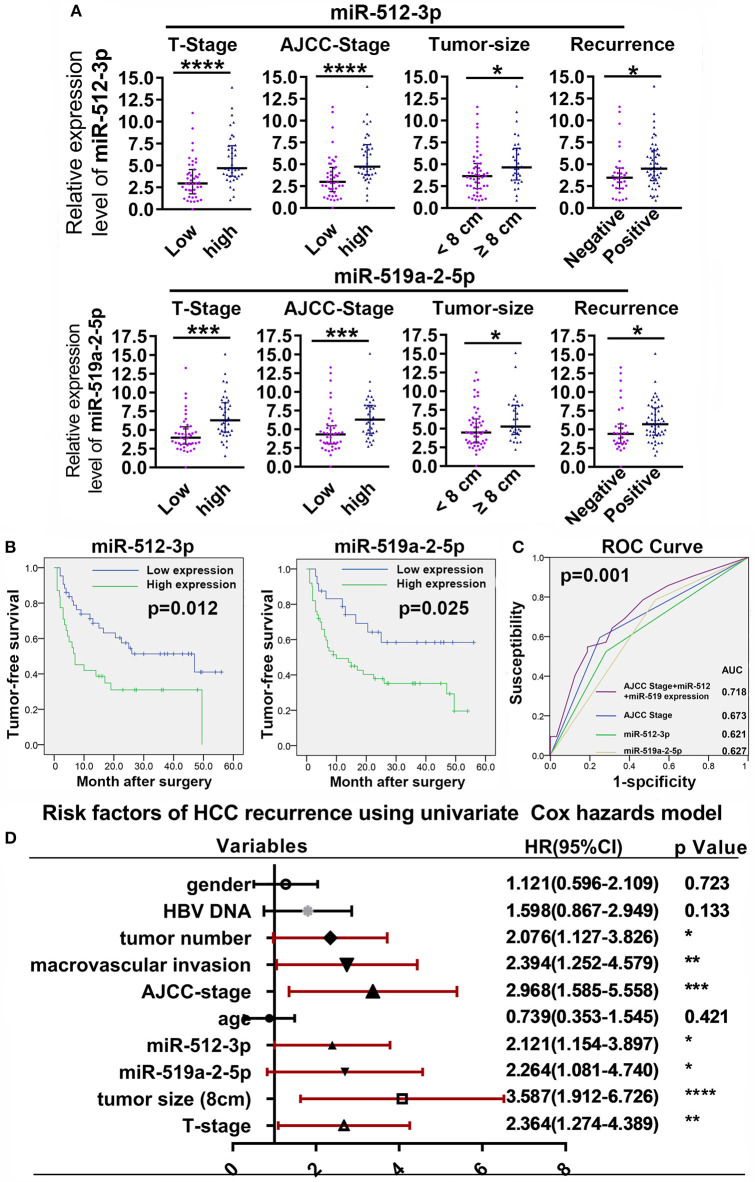
High expression of miR-519a-2-5p and miR-512-3p predicted early recurrence of HCC. **(A)** High T-Stage, high AJCC Stage, large tumor size (> 8 cm), and early recurrence were positively correlated with high miR-512-3p (upper layer) and miR-519a-2-5p expression (lower layer). Error bars represented the median ± interquartile range and *p*-values were calculated with the Mann–whiney test. **(B)** The comparison of cumulative tumor-free survival curves between the high and low expression of miR-512-3p (left) and miR-519a-2-5p (right). **(C)** ROC showed the prediction of HCC recurrence after the combination of miR-512-3p and miR-519a-2-5p. **(D)** Cox regression analysis showed the risk factors related to HCC early recurrence. “*”, “**”, “***” and “****” represented the *p*-value < 0.05, 0.01, 0.001 and 0.0001, respectively.

To evaluate the clinical value of the miR-519a-2-5p and miR-512-3p, the relative expression levels of the two miRNAs were detected from the tumor samples of 82 HCC patients, by q-PCR. The characteristics of the HCC patients were described in Table S2. The optimal cutoff values of the two miRNAs expression levels were determined by the ROC curves. The correlations between the clinicopathological characteristics and the two miRNAs were shown in Table S3. The results revealed that the expression of the two miRNAs was associated with the T Stage, AJCC Stage, tumor size, and tumor recurrence. Then, the patients of higher T Stage, higher AJCC Stage, larger tumor size, and the status of tumor recurrence presented higher expression of the two miRNAs (Figure 6A). Those results were also highly consistent with the conclusion of the *in vitro* and *in vivo* experiments in which the two miRNAs promoted HCC proliferation, invasion, and distant metastasis.

Compared with the patients with low expression of the miR-519a-2-5p and miR-512-3p, the patients with high expression of miR-519a-2-5p (*p* = 0.012) and miR-512-3p (*p* = 0.025) showed significantly poorer tumor-free survival (Figure 6B). To detect the predictability of HCC recurrence, the AUROC of AJCC-Stage was calculated as 0.673. After combining the two miRNAs with AJCC-Stage, the AUROC significantly elevated to 0.718 (*p* = 0.001), which indicated that the high expression of two miRNAs promoted the prediction of HCC recurrence (Figure 6C). The univariate Cox analyses demonstrated that the miR-519a-2-5p (HR=2.121, 95% CI=1.154–3.897, *p* = 0.015) and miR-512-3p (HR=2.264, 95%C I=1.081–4.740, *p* = 0.030), as well as tumor size, AJCC-Stage, T-Stage, macrovascular invasion were the significant risk factors of HCC recurrence (Figure 6D).

## Discussion

The tumor-associated miRNAs, which are considered as potential targets for cancer therapy, receive attention in the past two decades (23, 24). The principles for us to screen those two miRNAs as reasonable candidates as follow: (1) By digging the TCGA database before, we found that the two precursor miRNAs belonging to C19MC were upregulated and were positively associated with poor prognosis in HCC. (2) Compared with their complementary strands, the high levels of these two miRNAs in HCC proved that they are undoubtedly the dominant chain. (3) Besides, the expression levels of these two miRNAs are upregulated in our Chinese HCC cohorts, compared with the paraneoplastic tissues. (4) Through bioinformatics analysis, the two miRNAs may have potential common targets.

The function of miRNA clusters characterized by co-expression and similar effects is often powerful, and the similar regulation mechanisms on tumorigenesis and tumor development are also reported frequently (25, 26). The C19MC, a primate-specific miRNA cluster, which is located on chromosome 19q13.4, is the largest cluster that has been found (11). Through the current bioinformatics literature, C19MC showed consistent upregulation in multiple tumor types, which indicated that the co-upregulated expression of C19MC is highly correlated with tumorigenesis (27, 28). Interestingly, the ability of C19MC on promoting the invasion and metastasis of HCC particularly deserved attention. Four members of C19MC, miR-517a, miR-520c, miR-519d, miR-519c-3p, acted as oncogenes and pro-invasive roles on HCC, which were proved by Toffanin et al. (29) and Fornari et al. (30). In this study, by *in vivo* and *in vitro* experiments, we confirmed the similarity of miR-512-3p and miR-519a-2-5p in oncogenic property, especially on the aspect of promoting the invasion and distant metastasis of HCC, which validate the result of our previous bioinformatics analysis. If note, from our results, the effects of the two miRNAs on *in vivo* proliferation and metastasis are extremely similar. The speculation that all the upregulated C19MC members in HCC may have similar regulatory effects which needs to be further confirmed.

MAP3K2 and MAP2K4, two members of the JNK axis, show a sequential function of upstream and downstream in tumor regulation (22). Several reports have revealed that MAP3K2 and MAP2K4 in some tumor types showed tumor-suppressive effects, particularly on tumor invasion (31–33). In our study, we confirmed that they were tumor suppressor genes that were down-regulated in HCC, and our rescue experiments also demonstrated that miR-512-3p and miR-519a-2-5p promoted oncogenic effects by targeting the expression of MAP3K2 and MAP2K4.

Tumor recurrence after surgery remains the lethal issue of HCC. The 5-year tumor-free survival rate of HCC after surgery is only 27.7% (34). Xu et al. have presented that even the late recurrence (after 2 years) of HCC after surgery reaches 41.3% (35). The detection of miRNAs is significant for the prediction of tumor recurrence, which has been highlighted by intensive researches (36, 37). Our study sheds light on the significance of miR-519a-2-5p and miR-512-3p toward the HCC recurrence prediction. High expression of miR-519a-2-5p and miR-512-3p is positively associated with the key factors (AJCC Stage, T-Stage, and tumor size) of HCC and early recurrence of HCC after surgery. Besides, the combination of them improves the accuracy of the recurrence prediction of HCC. These results indicate that the two miRNAs can be superior biomarkers for the prediction of HCC recurrence.

This study existed limitations. The two miRNAs expression levels were detected from the tumor specimens. Further study should be demonstrated about liquid biopsy from patients' serum for evaluating the value of these two miRNAs in the diagnosis and prognosis of HCC.

## Conclusion

**Figure 7:**
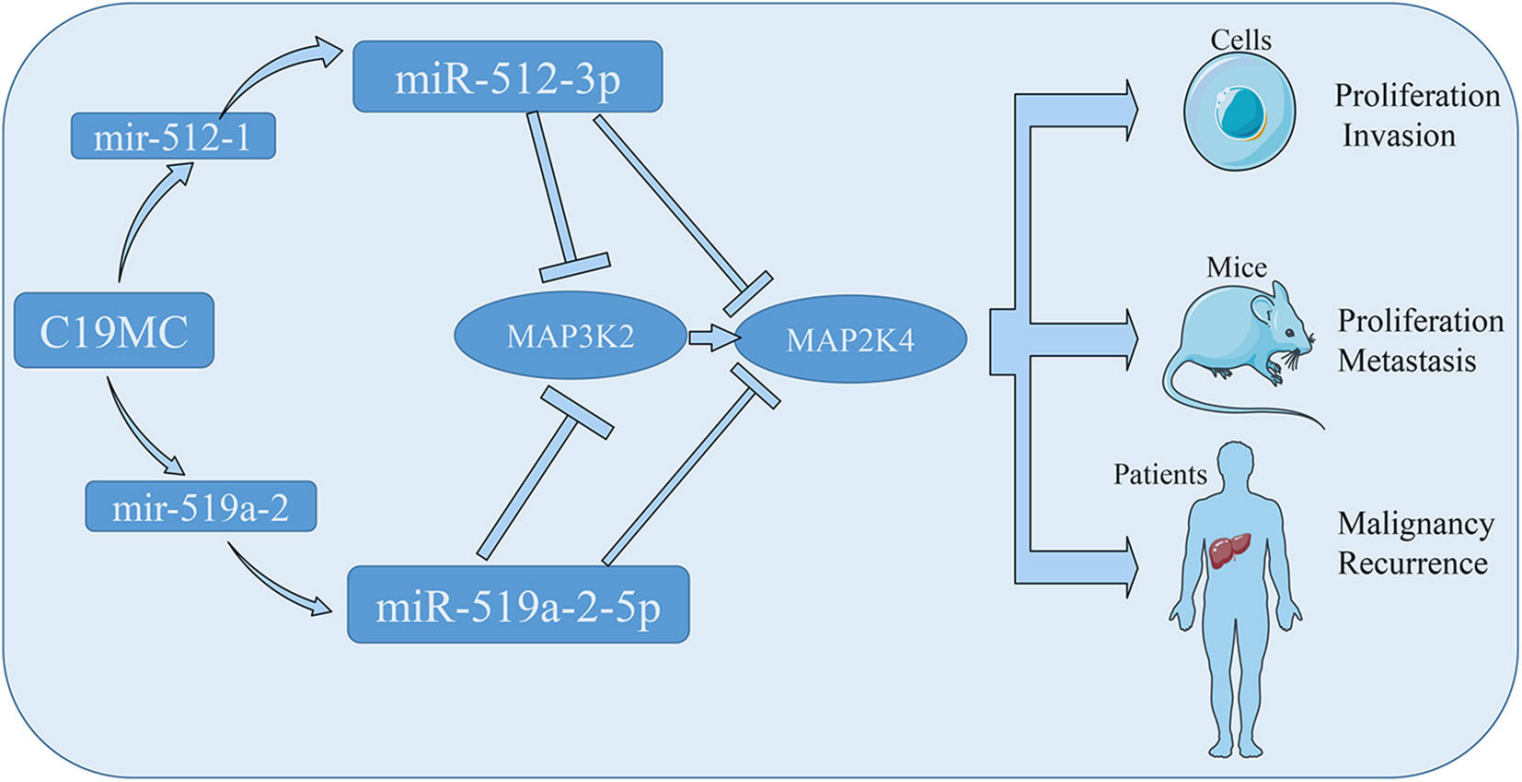
The schematic diagram showed that the miR-512-3p and miR-519a-2-5p promoted hepatocellular carcinoma progression by co-targeting the MAP3K2 and MAP2K4.

To conclude, we assessed two members of C19MC, miR-512-3p and miR-519a-2-5p, for the consistency of oncogenic property in HCC. The tumor suppressor genes, MAP3K2 and MAP2K4, were the direct targets, which were inhibited by the two miRNAs. The miR-512-3p and miR-519a-2-5p, promoted the HCC malignancy and predicted HCC early recurrence (Figure 7). All data suggested that they could be the novel targets for HCC therapy and significant markers for the prediction of HCC recurrence.

## Data Availability Statement

All datasets generated for this study are included in the article/Supplementary Material.

## Ethics Statement

The studies involving human participants were reviewed and approved by the Ethical Committee of the First Affiliated Hospital, Zhejiang University School of Medicine. The patients/participants provided their written informed consent to participate in this study. The animal study was reviewed and approved by the Ethical Committee of the First Affiliated Hospital, School of Medicine, Zhejiang University. Written informed consent was obtained from the owners for the participation of their animals in this study.

## Author Contributions

TR and QL performed the study. SZhe organized the study and provide funding support. TR and XZ performed most of the experiments. SF participated in immunohistochemistry. HX and LZ contributed to the collection and analysis of clinical information. TR, QL, SZha, and HH finished the statistical analysis. TR and QL prepared the draft. SZhe reviewed the manuscript. All authors have approved the final manuscript.

## Conflict of Interest

The authors declare that the research was conducted in the absence of any commercial or financial relationships that could be construed as a potential conflict of interest.

## Abbreviations

HCC: hepatocellular carcinoma
C19MC: chromosome 19 microRNA cluster
pre-mirna: precursor-miRNA
miRNA: mature miRNA
MAP3K2: mitogen-activated protein kinase 2
MAP2K4: mitogen-activated protein kinase kinase 4
TCGA: The Cancer Genome Atlas
JNK: c-Jun N-terminal kinase
MAPK: mitogen-activated protein kinase
KEGG: Kyoto Encyclopedia of Genes and Genomes
PPI: protein-protein interaction
SD: standard deviation
IQR: interquartile range
AUROC: area under receiver operating characteristics
3′UTR: 3′ untranslated region.
