# Supplementary material for: The Similar Effects of miR-512-3p and miR-519a-2-5p on the Promotion of Hepatocellular Carcinoma: Different Tunes Sung With Equal Skill
Source: Front Oncol. 2020 Aug 7;10:1244. doi: 10.3389/fonc.2020.01244 (PMC7427533; doi:10.3389/fonc.2020.01244)
Supplement: Supplementary file 1 [file Data_Sheet_1.docx]

**Fig.S1**


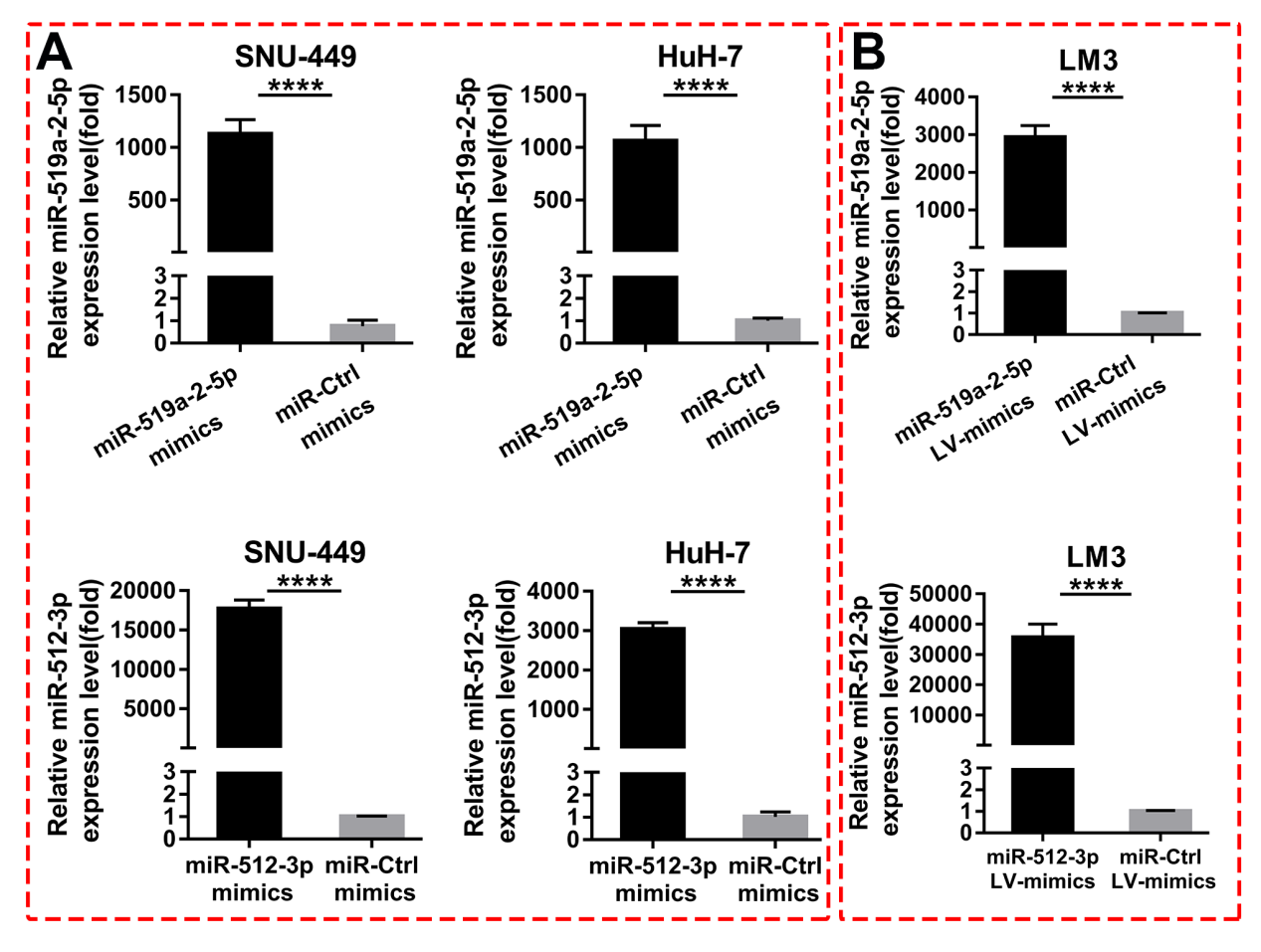


**Fig.S1** **A:** qRT-PCR assessed that the expression of miR-519a-2-5p or miR-512-3p was dramatically upregulated after the SNU-449, HUH-7 was transfected with miR-519a-2-5p or miR-512-3p mimics, respectively. **B:** qRT-PCR assessed that the LM3 cells stably expressed miR-519a-2-5p or miR-512-3p after the LM3 was transfected with miR-519a-2-5p or miR-512-3p lentiviral mimics. The statistics analysis was performed with the Mann-Whitney test.

**Fig.S2**


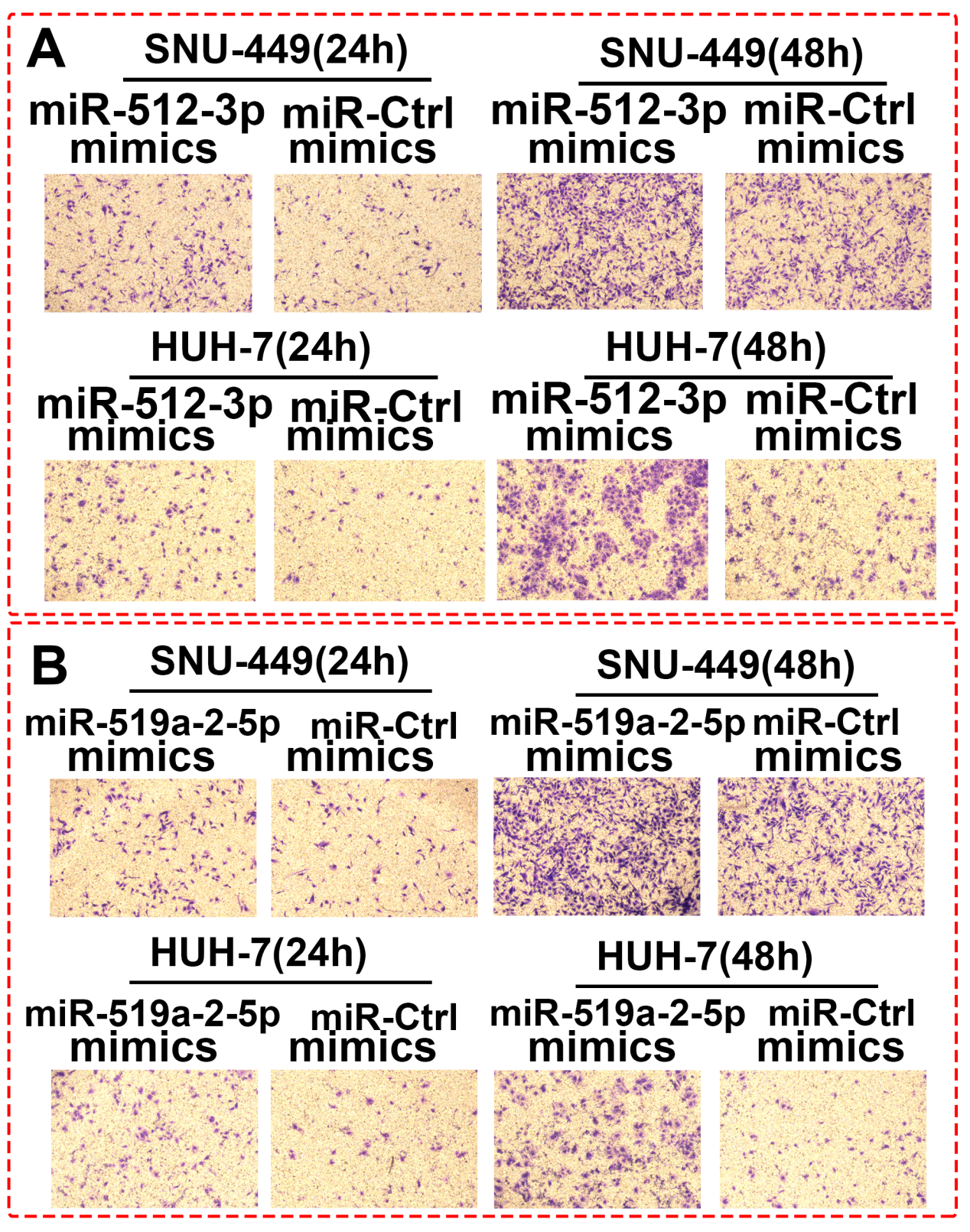


**Fig.S2** The images of transwell assays showed that the invasiveness (at time of 24h and 48h) of SNU-449 and HUH-7 after transfected with miR-512-3p (A) or miR-519a-2-5p (B) mimics and the controls, respective.

**Fig.S3**


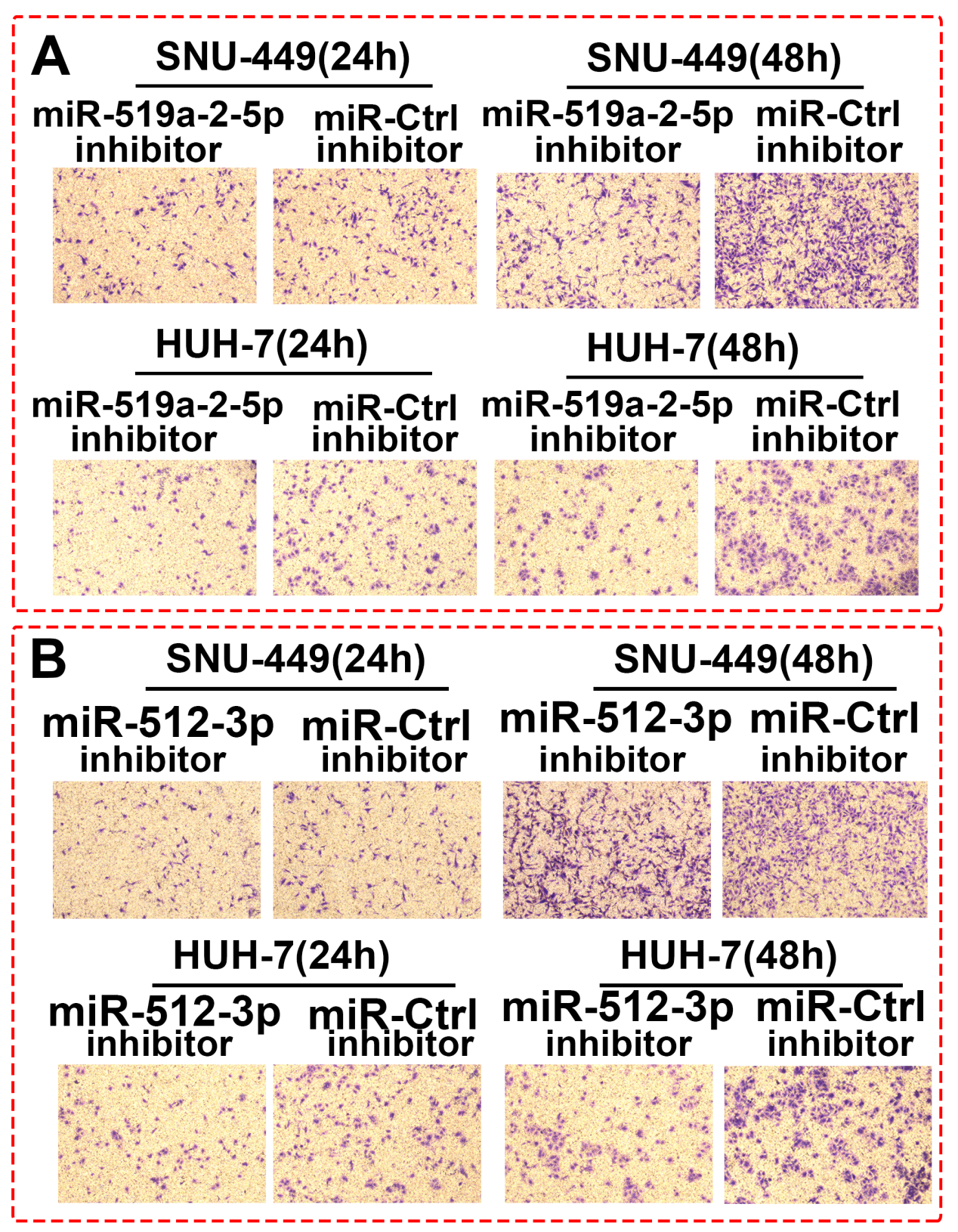
**Fig.S3** The images of transwell assays showing the invasiveness (at time of 24h and 48h) of SNU-449 and HUH-7 transfected with miR-512-3p (A) or miR-519a-2-5p (B) inhibitor and the controls, respective.

**Fig.S4**


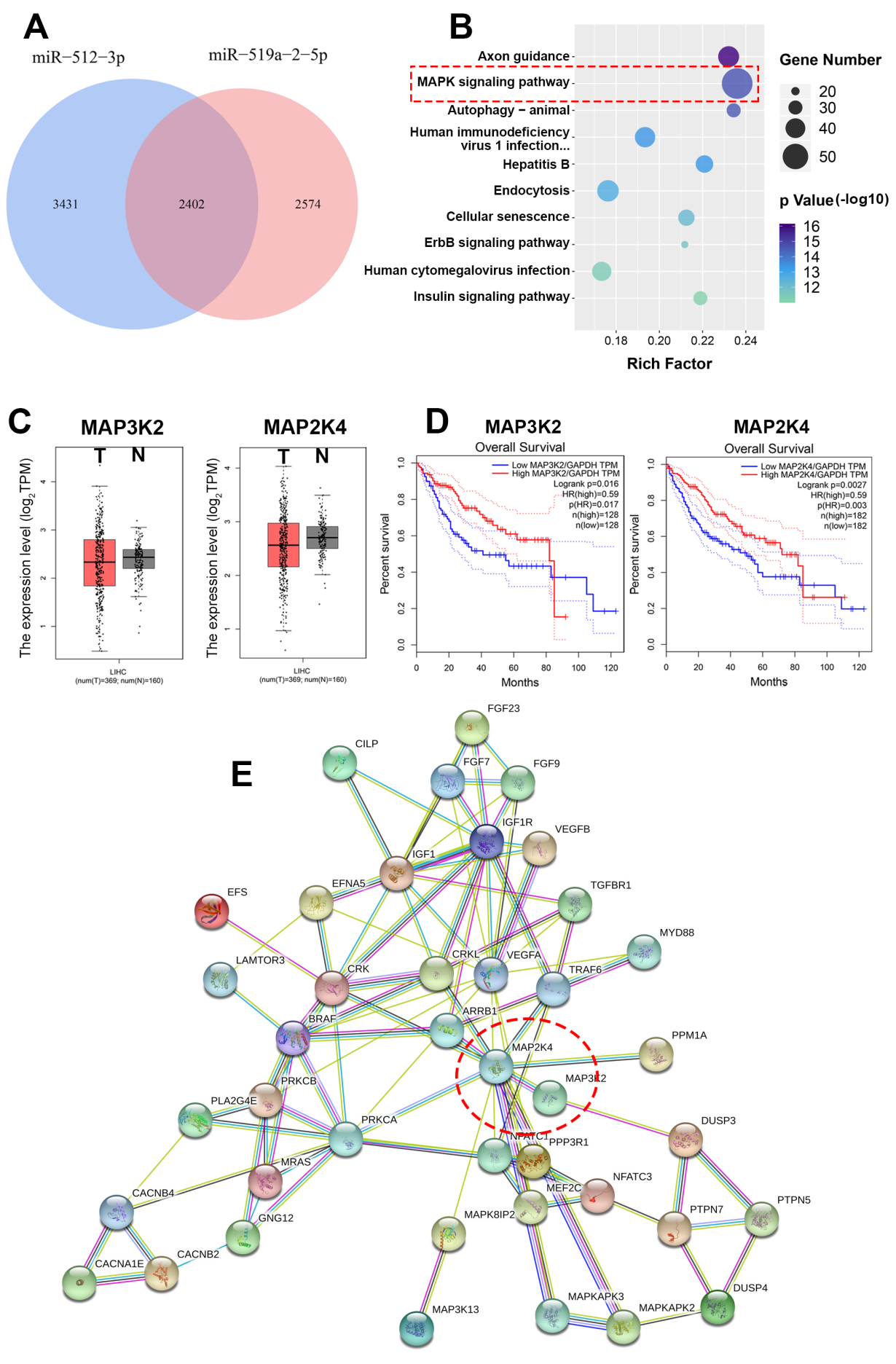


**Fig.S4:** (**A**) The Venn diagram showed the common targets of the two miRNAs. (**B**) The bubble chart showed the top 10 significant items which were from the KEGG enrichment of the common targets. (**C**) The expression levels of the MAP3K2 and MAP2K4 were shown, from the database of GEPIA. (**D**) The survival curves of the MAP3K2 and MAP2K4 were shown, from the database of GEPIA. (**E**) The PPI network of the potential targets was constructed. The MAP3K2 and MAP2K4 were at the core of the targets construction network.

**Fig.S5**


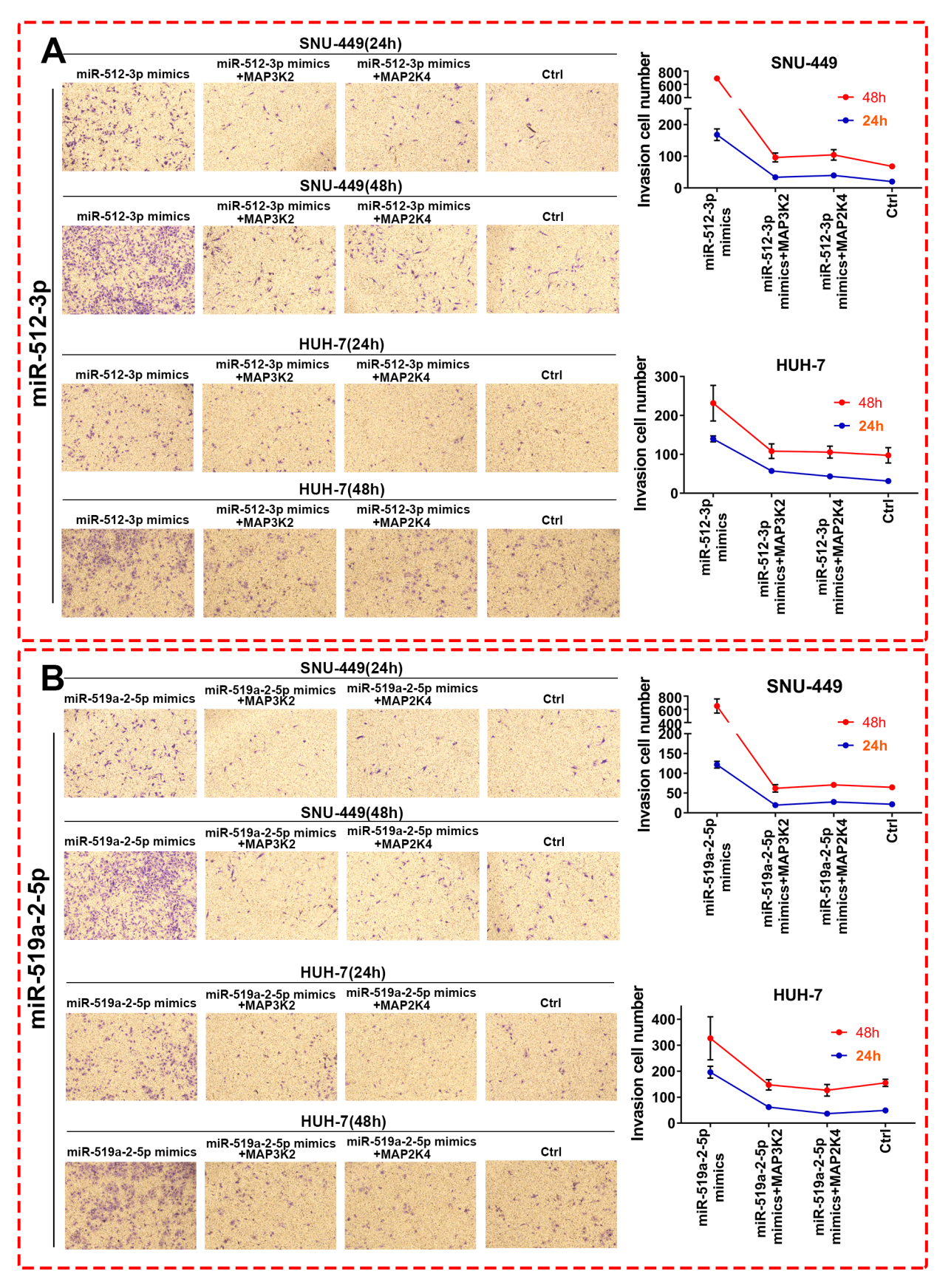


**Fig.S5.A-B** Upregulation of MAP3K2 or MAP2K4 reversed the invasiveness promotion of the miR-512-3p or miR-519a-2-5p. Images and line charts of transwell assays showed the change of invasiveness in SNU-449 and HUH-7 which are co-transfected with miR-512-3p (A) or miR-519a-2-5p (B) mimics and MAP3K2 or MAP2K4 plasmid, compared with their controls.

**Fig.S6**


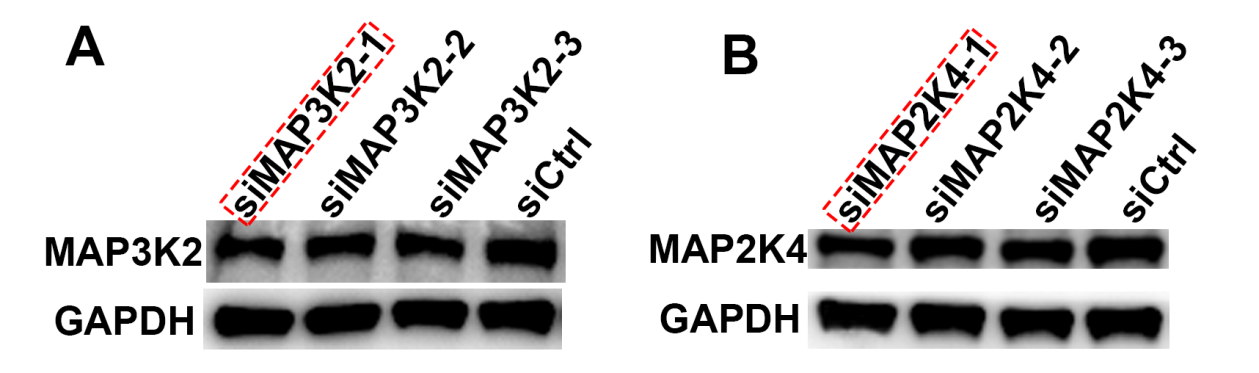


**Fig.S6**.**A-B:** Western blot analysis of the MAP3K2 (A) or MAP2K4 (B) after SNU-449 was transfected with three MAP3K2 siRNA candidates or MAP2K4 siRNA candidates. The siMAP3K2-1 and the siMAP2K4-1 were confirmed as the most significant.

**Fig.S7**


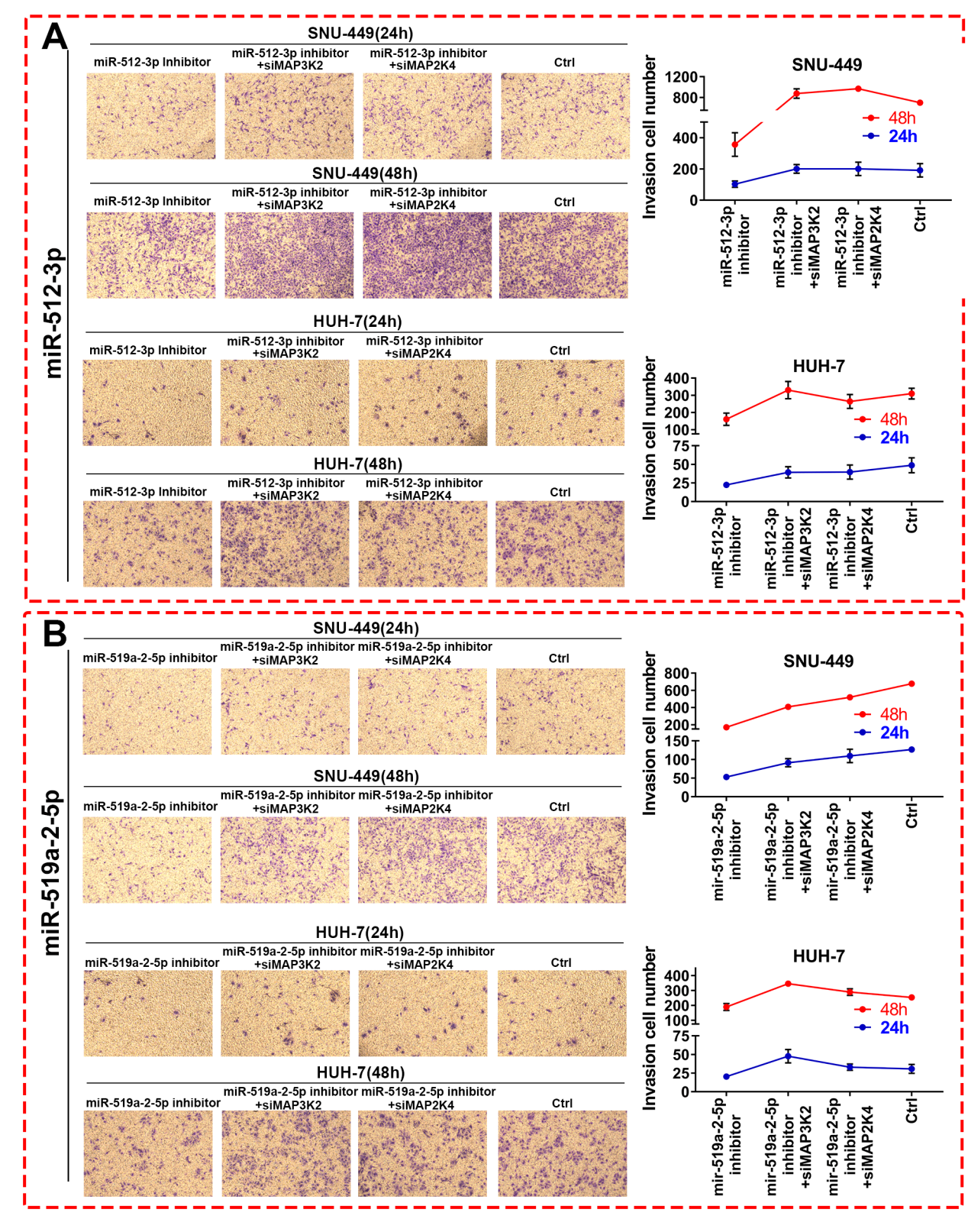


**Fig.S7.A-B** MAP3K2 or MAP2K4 Knockdown reversed the invasion depression of the miR-512-3p or miR-519a-2-5p inhibitor. Images and line charts of transwell assays showed the change of invasiveness in SNU-449 and HUH-7 which are co-transfected with miR-512-3p (A) or miR-519a-2-5p (B) inhibitor and MAP3K2 or MAP2K4 siRNA, compared with their controls.

**Table.S1 The primers sequences for mRNA amplification.**

| Name | Sequence |
| --- | --- |
| MAP3K2 For | TGATCTGTTTTATCTTCTCAGGCCA |
| MAP3K2 Rev | TGATCATCCATTATGGCAAACAGC |
| MAP2K4 For | CAGGAGTTCAAAACCCACACA |
| MAP2K4 Rev | ATCCCAGTGTTGTTCAGGGG |
| GAPDH For | GCACCGTCAAGGCTGAGAAC |
| GAPDH Rev | TGGTGAAGACGCCAGTGGA |

**Table.S2 Clinical characteristics for the 82 HCC patients**

| Factor | Mean ± SD |  | N | % |
| --- | --- | --- | --- | --- |
| Age (years) | 54.6 ± 12.9 |  |  |  |
|  |  | ≥ 65 | 60 | 73.2% |
|  |  | < 65 | 22 | 26.8% |
| Gender |  | Female | 28 | 34.1% |
|  |  | Male | 54 | 65.9% |
| Tumor size (cm) | 8.5 ± 4.2 |  |  |  |
|  |  | ≥ 8 | 51 | 62.2% |
|  |  | < 8 | 31 | 37.8% |
| T Stage |  | Low (stage I+II) | 46 | 56.1% |
|  |  | High (stage III+Ⅳ) | 36 | 43.9% |
| Lymph node metastasis |  | Negative | 75 | 91.5% |
|  |  | Positive | 7 | 8.5% |
| AJCC Stage |  | Low (stage I+II) | 44 | 53.7% |
|  |  | High (stage III+Ⅳ) | 38 | 46.3% |
| Macrovascular invasion |  | Negative | 59 | 72.0% |
|  |  | Positive | 23 | 28.0% |
| Tumor differentiation |  | Grade Ⅰ | 9 | 11% |
|  |  | Grade Ⅱ | 40 | 48.8% |
|  |  | Grade Ⅲ | 33 | 40.2% |
| Tumor number |  | Isolated | 54 | 65.9% |
|  |  | Multiple | 28 | 34.1% |
| Tumor location |  | Right lobe | 59 | 72.0% |
|  |  | Left lobe | 17 | 20.7% |
|  |  | Bilateral lobe | 6 | 7.3% |
| miR-512-3p expression |  | High | 37 | 45.1% |
|  |  | Low | 45 | 54.9% |
| miR-519a-2-5p expression |  | High | 56 | 68.3% |
|  |  | Low | 26 | 31.7% |
| Cirrhosis |  | Negative | 19 | 23.2% |
|  |  | Positive | 63 | 76.8% |
| Child-Pugh class |  | Grade A | 81 | 98.8% |
|  |  | Grade B | 1 | 1.2% |
| HBV DNA |  | > 10^3^ | 38 | 46.4% |
|  |  | ≤ 10^3^ | 44 | 53.7% |
| HBsAg |  | Positive | 67 | 81.7% |
|  |  | Negative | 15 | 18.3% |
| Tumor Recurrence |  | Positive | 42 | 56.8% |
|  |  | Negative | 32 | 43.2% |
| AFP (>20ng/ml) |  | Positive | 48 | 59.3% |
|  |  | Negative | 33 | 40.7% |
| AFP |  | >100 ng/ml | 34 | 42.0% |
|  |  | ≤100 ng/ml | 47 | 58.0% |
| AFP |  | >400 ng/ml | 25 | 39.1% |
|  |  | ≤400 ng/ml | 56 | 60.1% |
| Albumin (g/L) | 43.2 ± 4.0 |  |  |  |
| Prothrombin time (S) | 11.9 ± 1.2 |  |  |  |
| INR | 1.04 ± 0.1 |  |  |  |
| Total bilirubin (μmol/L) | 14.8 ± 7.0 |  |  |  |
| ALT (U/L) | 33.9 ± 20.1 |  |  |  |
| AST (U/L) | 45.9 ± 32.6 |  |  |  |
| Creatinine (μmol/L) | 71.5 ± 13.2 |  |  |  |
| Leukocyte (×10^9^/L) | 5.8 ± 2.1 |  |  |  |
| Platelet (×10^9^/L) | 179.6 ± 59.4 |  |  |  |
| Hemoglobin (g/L) | 141.8 ± 17.2 |  |  |  |

Abbreviations: HCC: hepatocellular carcinoma; AFP: α-fetoprotein; INR: international normalized ratio; Abbreviations: ALT: alanine aminotransferase; AST: alanine aminotransferase; HBsAg: hepatitis B Surface Antigens.

**Table.S3** The correlation between the two miRNAs and clinicopathological characteristics.

| Charactristics | miR-512-3p (n) | | |  | miR-519a-2-5p (n) | | | |  |
| --- | --- | --- | --- | --- | --- | --- | --- | --- | --- |
|  | Low | High | p |  | Low | High | p | |  |
| Age (years) |  |  |  |  |  |  |  | |  |
| <65 | 31 | 29 | 0.334 |  | 20 | 40 | 0.601 | |  |
| ≥65 | 14 | 8 |  |  | 6 | 16 |  | |  |
| Gender |  |  |  |  |  |  |  | |  |
| Female | 15 | 13 | 0.864 |  | 7 | 21 | 0.347 | |  |
| Male | 30 | 24 |  |  | 19 | 35 |  | |  |
| **T Stage** |  |  |  |  |  |  |  | |  |
| Low (stage I+II) | 32 | 14 | 0.003 |  | 22 | 24 | < 0.001 | |  |
| High (stage III+Ⅳ) | 13 | 23 |  |  | 4 | 32 |  | |  |
| **AJCC Stage** |  |  |  |  |  |  |  | |  |
| Low (stage I+II) | 31 | 13 | 0.002 |  | 20 | 24 | 0.004 | |  |
| High (stage III+Ⅳ) | 14 | 24 |  |  | 6 | 32 |  | |  |
| **Tumor Size** |  |  |  |  |  |  |  | |  |
| < 8cm | 32 | 19 | 0.066 |  | 20 | 31 | 0.061 | |  |
| ≥ 8cm | 13 | 18 |  |  | 6 | 25 |  | |  |
| **Tumor Recurrence** |  |  |  |  |  |  | |  | |
| Positive | 23 | 9 | 0.036 |  | 15 | 17 | 0.021 | |  |
| Negative | 20 | 22 |  |  | 9 | 33 |  | |  |
| Lymph node metastasis |  |  |  |  |  |  |  | |  |
| Negative | 43 | 32 | 0.235 |  | 25 | 50 | 0.422 | |  |
| Positive | 2 | 5 |  |  | 1 | 6 |  | |  |
| Tumor number |  |  |  |  |  |  |  | |  |
| Isolated | 33 | 21 | 0.115 |  | 22 | 32 | 0.023 | |  |
| Multiple | 12 | 16 |  |  | 4 | 24 |  | |  |
| Macrovascular invasion |  |  |  |  |  |  |  | |  |
| Negative | 35 | 24 | 0.195 |  | 21 | 38 | 0.226 | |  |
| Positive | 10 | 13 |  |  | 5 | 18 |  | |  |
| HBsAg positive |  |  |  |  |  |  |  | |  |
| Positive | 8 | 7 | 0.894 |  | 5 | 10 | 0.881 | |  |
| Negative | 37 | 30 |  |  | 21 | 46 |  | |  |
| HBV DNA |  |  |  |  |  |  |  | |  |
| > 10^3^ | 24 | 20 | 0.948 |  | 13 | 31 | 0.651 | |  |
| ≤ 10^3^ | 21 | 17 |  |  | 13 | 25 |  | |  |
| AFP |  |  |  |  |  |  |  | |  |
| >20 ng/ml | 18 | 15 | 0.973 |  | 13 | 20 | 0.169 | |  |
| ≤20 ng/ml | 26 | 22 |  |  | 12 | 36 |  | |  |
| >100 ng/ml | 26 | 21 | 0.832 |  | 17 | 30 | 0.224 | |  |
| ≤100 ng/ml | 18 | 16 |  |  | 8 | 26 |  | |  |
| >400 ng/ml | 30 | 26 | 0.839 |  | 21 | 35 | 0.070 | |  |
| ≤400 ng/ml | 14 | 11 |  |  | 4 | 21 |  | |  |

Abbreviations: HBsAg: hepatitis B Surface Antigens; AFP: α-fetoprotein.
